# Heat-Induced HSAT3 Transcription and Nuclear Stress Body Assembly in Human Myeloid Cells

**DOI:** 10.3390/ijms27209146

**Published:** 2026-10-15

**Authors:** Nikita Ponomartsev, Natella Enukashvily

**Affiliations:** Institute of Cytology, Russian Academy of Sciences, St. Petersburg 194064, Russia

**Keywords:** non-coding RNA, human satellite 3, nuclear stress bodies, heat shock, cell stress, HSF1

## Abstract

Heat shock activates heat shock factor 1 (HSF1), which induces classical heat shock genes and promotes transcription of pericentromeric HSAT3 repeats that contribute to nuclear stress body (nSB) formation. Although well characterized in epithelial cells, this response remains poorly understood in myeloid cells. We compared heat-induced HSAT3 transcription and nSB assembly in epithelial and monocytic cell lines, using A549 and THP-1 as models. pHSF1 (Ser326) levels, *HSPA1A* and HSAT3 expression, and nSB formation were assessed by Western blotting, qPCR, immunofluorescence, RNA-FISH, and 5-ethynyluridine labeling. Heat shock increased pHSF1 (Ser326) levels and *HSPA1A* expression in both models. A549 cells showed increased HSAT3 transcript levels and prominent nSB formation, whereas undifferentiated THP-1 cells showed little immediate change in HSAT3 transcript levels and did not form prominent nSBs. 5-EU labeling revealed discrete nascent RNA foci in heat-shocked A549, but not THP-1, cells. These foci overlapped with HSF1 and were associated with HSAT3-related hybridization signals. However, click chemistry increased accessibility of genomic satellite sequences, limiting RNA-specific interpretation of the HSAT3 probe signal. PMA-induced differentiation enabled an increase in HSAT3 transcript levels during heat shock and nSB assembly. These findings identify cellular state as a determinant of heat shock-induced satellite transcription and nSB assembly.

## 1. Introduction

Heat shock is one of several universal stressors that trigger a highly conserved cellular response to maintain proteostasis and preserve cell viability. Heat shock factor 1 (HSF1) is the central regulator of this response; it is activated by increased temperature, undergoes post-translational modifications, and induces transcription of genes that protect cells from stress-induced damage [1]. In addition to regulating the transcription of classic heat shock genes, HSF1 can also control the transcription of non-coding repetitive genomic elements, including pericentromeric satellite sequences [2].

Satellite DNA is a class of highly repetitive nucleotide sequences arranged in tandem that are predominantly located in heterochromatic regions such as centromeres and pericentromeres [3]. Centromeric and pericentromeric satellite DNA were long considered to be primarily structural components of constitutive heterochromatin that provided architectural organization of chromosomes. Among human satellite repeats, HSAT3 undergoes the strongest transcriptional activation in response to heat shock [4,5]. HSAT3 transcripts remain localized near their transcription sites and, by recruiting various RNA-binding proteins, initiate the formation of ribonucleoprotein (RNP) compartments known as nuclear stress bodies (nSBs). HSF1, which activates HSAT3 transcription, also colocalizes with these structures and serves as one of their primary markers [6]. The largest nSBs are localized in association with the pericentromeric regions of chromosomes 9 and Y, which have the largest HSAT3 clusters [7,8,9]. nSBs are thought to serve as temporary storage sites for nuclear proteins, especially splicing factors, as well as their associated transcripts, including unprocessed RNA. This sequestration is considered a protective mechanism that stabilizes these molecules and prevents their premature engagement in transcription and pre-mRNA splicing during stress-induced perturbation of cellular homeostasis. Upon recovery from stress and restoration of physiological conditions, nuclear stress bodies rapidly disassemble, leading to the release of their components. This is accompanied by the reactivation of the splicing machinery and the rapid restoration of normal gene expression [10]. In addition to the “reservoir” model, alternative functions of nSBs have been proposed. In particular, nSBs have been suggested to regulate the transcription of repetitive genomic regions, promote chromatin remodeling in pericentromeric regions, and coordinate stress-induced reorganization of nuclear architecture [6]. Thus, nSBs are increasingly recognized not only as transient storage sites for nuclear proteins and RNAs but also as dynamic regulatory platforms that contribute to the regulation of gene expression at both the transcriptional and post-transcriptional levels during the cellular stress response. Because nSB assembly depends on activation of pericentromeric satellite transcription, identifying the cellular contexts in which this pathway is activated is essential for understanding the physiological functions of these nuclear compartments.

Despite considerable progress in understanding stress-induced HSAT3 transcription and nSB formation, most studies have been limited to a small number of epithelial cell lines, predominantly HeLa cells [5,8,10,11,12,13,14]. Consequently, it remains unclear whether this response is conserved across cells of different tissue origins and how it is influenced by the functional state of the cell. Among non-epithelial cells, myeloid cells are of particular interest because they provide a valuable model for investigating HSAT3 transcription regulation, as their differentiation and polarization are accompanied by extensive epigenetic remodeling and changes in chromatin accessibility [15]. Such epigenetic changes may alter the transcriptional activity of pericentromeric repeats. This is particularly relevant given accumulating evidence implicating HSAT2/HSAT3 transcripts in inflammatory processes. Elevated expression of HSAT2/HSAT3 transcripts has been associated with a pro-inflammatory phenotype and the upregulation of cytokine and chemokine genes [16,17]. These observations suggest that HSAT3 transcription may play an important role in immune regulation and underscore the need to investigate this response in physiologically relevant cellular models. Therefore, this study aimed to determine whether heat-induced HSAT3 transcription and nSB assembly depend on cellular identity and differentiation state. We compared the heat shock response in epithelial and monocytic cell models and further examined whether macrophage-like differentiation alters HSAT3 responsiveness in monocytic cells. Our results show that activation of the canonical HSF1-mediated heat shock response can occur without concomitant HSAT3 induction or nSB formation in undifferentiated monocytic cells. In contrast, PMA-induced differentiation into macrophage-like cells enabled heat-induced HSAT3 transcription and nSB assembly, indicating that differentiation state is an important determinant of this branch of the heat shock response.

## 2. Results

### 2.1. Activation of HSF1 and Induction of HSPA1A Expression in Response to Hyperthermia

**Figure 1:**
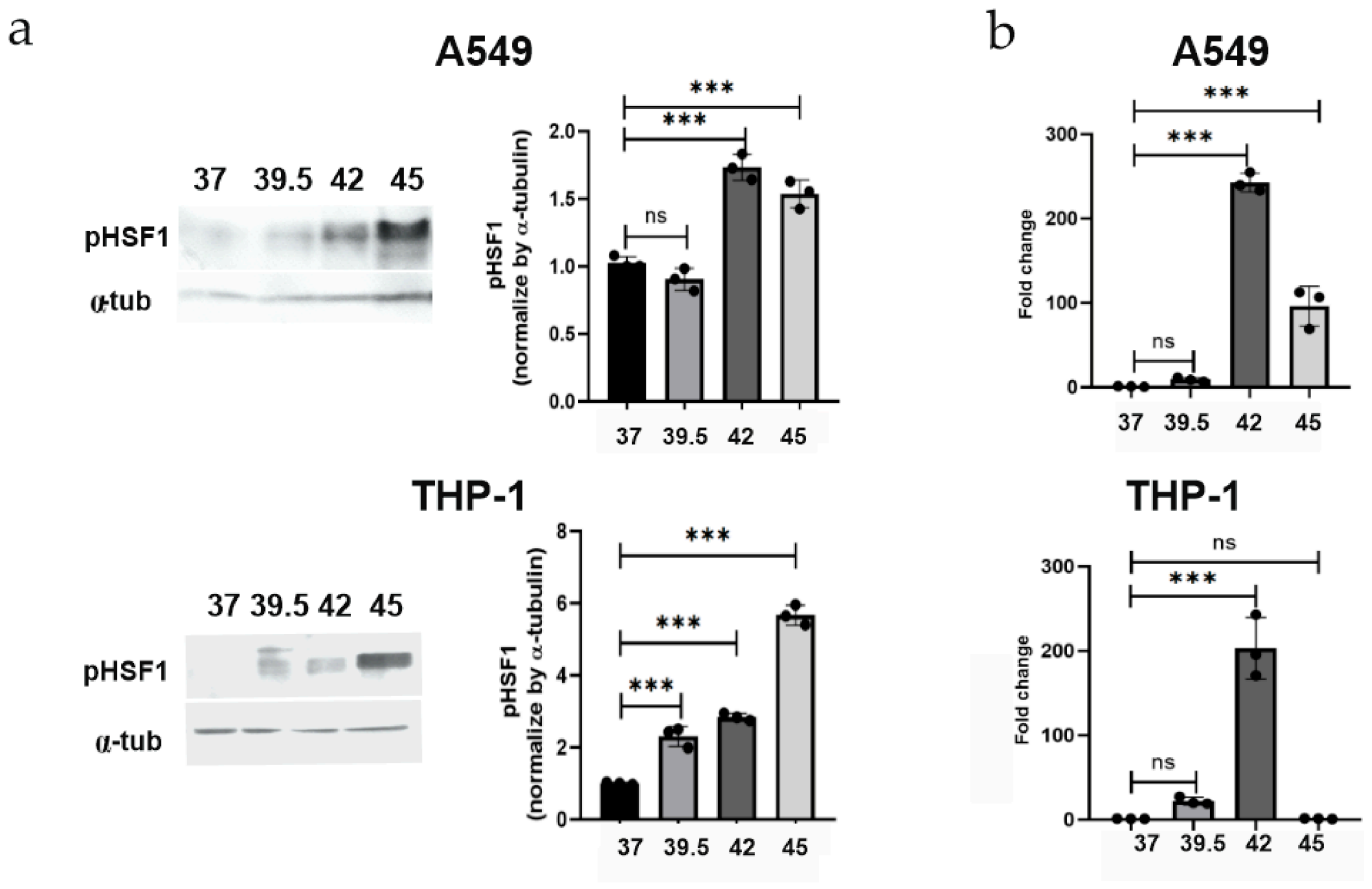
Temperature-dependent changes in pHSF1 (Ser326) levels and *HSPA1A* expression in A549 and THP-1 cells after 1 h of heat exposure. (**a**) Detection of pHSF1 in A549 and THP-1 cells by Western blotting. α-tubulin (α-tub) was used as a loading control. Corresponding densitometric quantification of pHSF1 normalized to α-tubulin is shown. (**b**) Relative *HSPA1A* mRNA expression levels in A549 and THP-1 cells determined by qPCR and normalized to *GAPDH*. Data are presented as mean ± SD. *** *p* < 0.001, ns, not significant compared with control cells (37 °C).

HSF1 is a key regulator of the cellular heat shock response, and its activation is accompanied by phosphorylation. Therefore, as a first step, we analyzed pHSF1 levels in cells exposed to different degrees of hyperthermia. Cells were exposed for 1 h to 39.5 °C, representing febrile-range hyperthermia, 42 °C, a commonly used experimental heat-shock condition, or 45 °C, representing severe heat stress. Cells maintained at 37 °C served as controls. The selected temperatures spanned a range of thermal stress, from febrile-range hyperthermia to severe heat stress [18,19]. In A549 cells, a statistically significant increase in pHSF1 levels was observed at 42 and 45 °C. In THP-1 cells, an increase in pHSF1 levels was detectable as early as 39.5 °C and rose as the temperature increased, reaching a maximum at 45 °C (Figure 1a). To assess HSF1 transactivation activity, we analyzed mRNA levels of *HSPA1A*, a canonical HSF1 target gene encoding inducible HSP70-1A. *HSPA1A* mRNA levels increased at 39.5 °C and reached maximal levels at 42 °C in both cell lines, while expression remained elevated at 45 °C but was significantly lower than that observed at 39.5 °C (Figure 1b).

### 2.2. Heat-Induced HSAT3 Transcription Differs Between Epithelial and Monocytic Cell Models

To investigate how cellular identity affects heat-induced satellite transcription, we assessed HSAT3 expression using qPCR and RNA-FISH. For qPCR, cDNA was amplified using the primers described by Valgardsdottir et al. [5], which were designed to amplify the HSAT3 array represented by the chromosome 9 reference sequence X06137.1. Thus, the qPCR assay primarily reflects transcription from this HSAT3 locus. For spatial visualization of HSAT3 transcripts, we performed RNA-FISH using the HSAT3 probe (DYZ1), which consists of tandem CCATT repeats that represent core sequence elements shared by HSAT3-related pericentromeric satellite repeats [9]. Under stringent hybridization and post-hybridization washing conditions, DYZ1 predominantly detects pericentromeric HSAT3 sequences on chromosomes Y and 9 [7]. In our previous work, we established less stringent hybridization and washing conditions under which the same probe produces signals in pericentromeric regions of multiple chromosomes [20]. These conditions were used in the present study to visualize a broader pool of HSAT3-containing transcripts originating from multiple chromosomal loci rather than being restricted to chromosomes Y and 9. This approach is relevant because heat-induced HSAT3 transcription has been reported from multiple pericentromeric loci containing HSAT3-related repeats [21]. Thus, qPCR provides a quantitative measure of HSAT3 transcription from the chromosome 9 locus, whereas RNA-FISH enables visualization of the subnuclear distribution and relative accumulation of HSAT3-related satellite transcripts originating from multiple pericentromeric loci.

**Figure 2:**
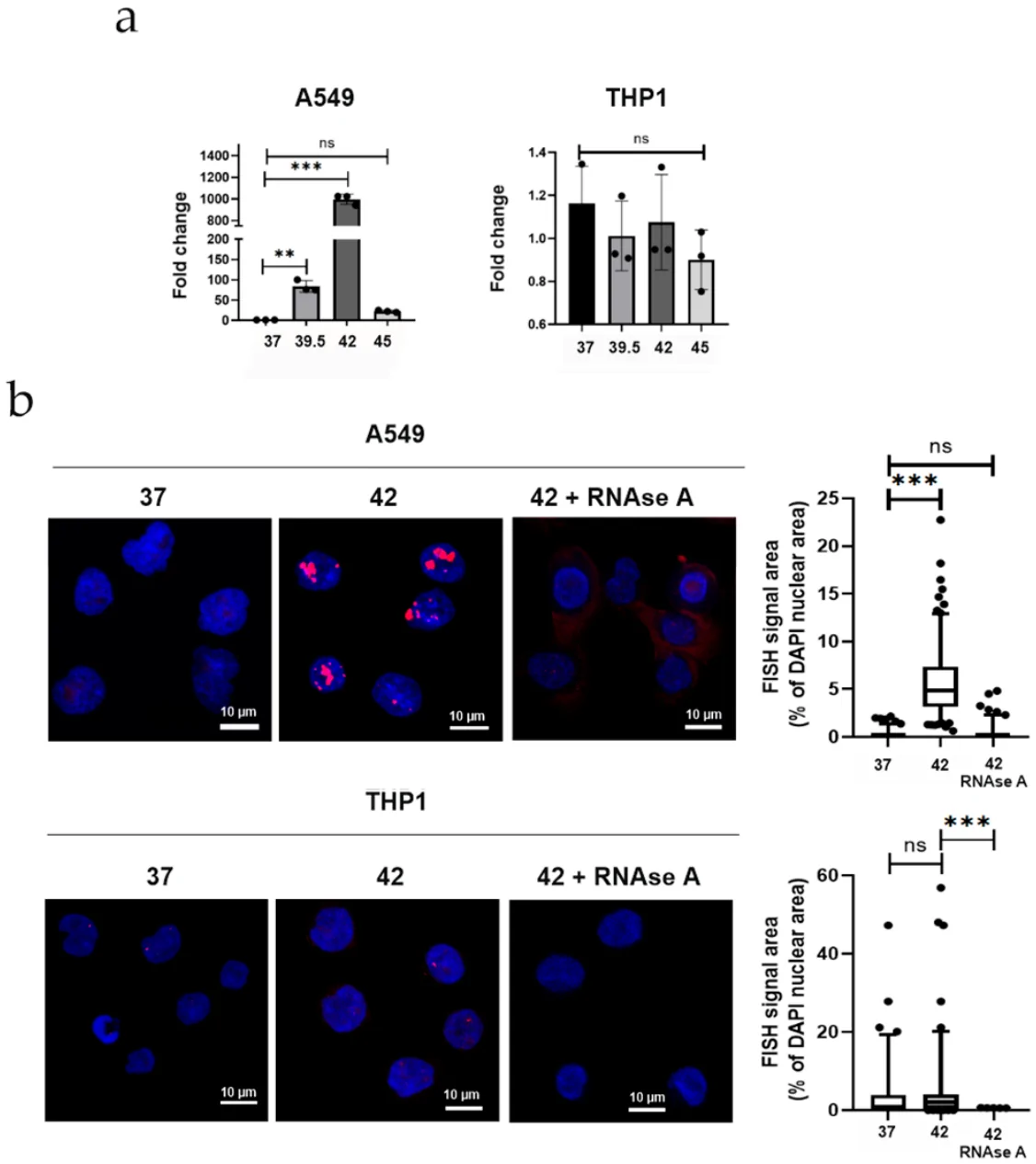
Heat-induced accumulation of HSAT3 transcripts in A549 but not THP-1 cells after 1 h of heat shock. (**a**) Relative HSAT3 transcript levels in A549 and THP-1 cells exposed to 39.5, 42, or 45 °C for 1 h. Cells maintained at 37 °C served as controls. Expression levels are presented as fold changes relative to the corresponding control. Data are presented as mean ± SD. (**b**) Representative RNA-FISH images showing HSAT3 transcripts (red) in A549 and THP-1 cells maintained at 37 °C, exposed to 42 °C for 1 h or exposed to 42 °C for 1 h followed by RNase A treatment before hybridization. Nuclei were counterstained with DAPI (blue). Scale bars, 10 μm. Scatter plots show the fraction of the nuclear area occupied by the RNA-FISH signal for individual cells. Boxes indicate the interquartile range, with the median shown as a horizontal line. ** *p* < 0.01, *** *p* < 0.001; ns, not significant.

In A549 cells, HSAT3 transcript levels measured by qPCR increased slightly following exposure to 39.5 °C and showed a pronounced induction after exposure to 42 °C, reaching more than a 1000-fold increase relative to control cells (Figure 2a). At 45 °C, HSAT3 transcript levels decreased compared with 42 °C and remained above control levels; however, the difference from 37 °C was not statistically significant. In contrast, no significant heat-induced increase in HSAT3 expression was detected in THP-1 cells. These results contrasted with activation of the canonical HSF1 pathway under the same experimental conditions. THP-1 cells exhibited increased pHSF1 (Ser326) levels and induction of the classical HSF1 target gene *HSPA1A* without a corresponding increase in HSAT3 transcription (Figure 1). A similar dissociation between the canonical heat shock response and HSAT3 activation was observed in another monocytic cell line, U937: heat shock induced *HSPA1A* expression, whereas HSAT3 transcription remained unchanged (Figure S1a). To determine whether HSAT3 induction in THP-1 cells might exhibit different kinetics, we additionally analyzed HSAT3 transcript levels during prolonged heat exposure and during the post-stress recovery period. Extending heat exposure to 2 h did not result in a marked increase in HSAT3 transcript levels. No marked increase was observed after 1 h of recovery; however, after 3 h of recovery following 1 h of heat shock at 42 °C, HSAT3 transcript levels showed a delayed increase, reaching approximately 3.6-fold relative to untreated cells (Figure S1b).

The RNA-FISH analysis was largely consistent with the qPCR data (Figure 2b). No specific HSAT3-related signal was detected in control A549 cells, whereas exposure to 42 °C resulted in prominent nuclear foci in the majority of cells. Quantitative image analysis demonstrated a significant increase in the fraction of nuclear area occupied by HSAT3-related satellite signals. RNase A treatment markedly reduced the HSAT3-related signal in heat-shocked A549 cells to levels that did not differ significantly from those in control cells, confirming the RNA-dependent nature of the detected foci. In THP-1 cells, weak punctate signals were detected under control conditions, but their nuclear area did not increase significantly following heat exposure. RNase A treatment also significantly reduced the residual HSAT3-related signal in heat-shocked THP-1 cells (Figure 2b).

Together, these findings show that the canonical HSF1-mediated heat shock response does not necessarily result in an immediate increase in HSAT3 transcript levels in different cell types.

### 2.3. Spatial Relationship Between Nascent RNA and HSAT3 Signals After Heat Shock

**Figure 3:**
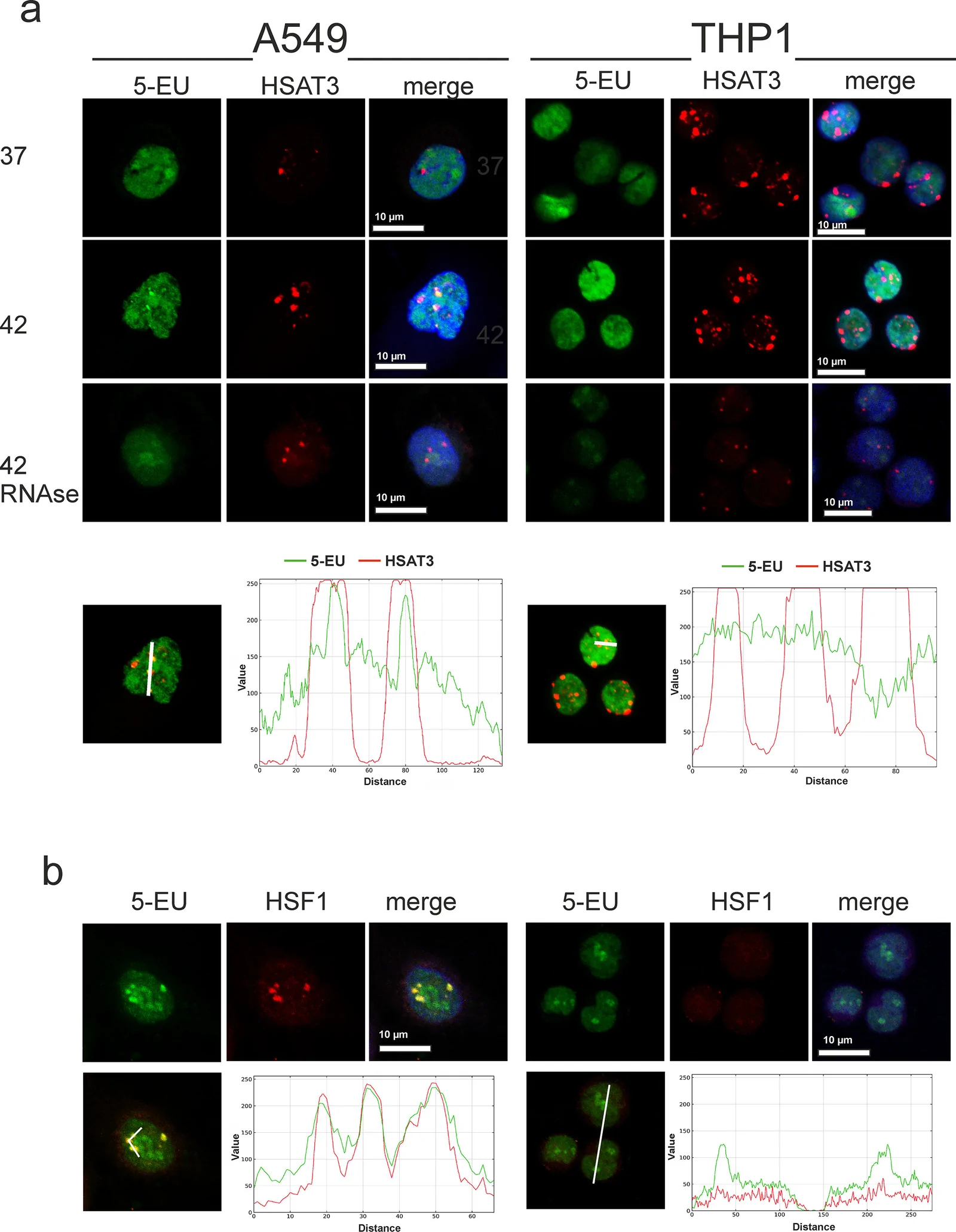
Spatial relationship between nascent RNA, HSAT3-related satellite signals, and HSF1 in A549 and THP-1 cells following heat shock. (**a**) Representative images of A549 and THP-1 cells incubated with 5-ethynyluridine (5-EU) during heat treatment and maintained at 37 °C, exposed to 42 °C for 1 h, or exposed to 42 °C for 1 h followed by RNase A treatment before hybridization. Newly synthesized RNA was labeled by 5-EU incorporation and visualized by Cu(I)-catalyzed azide–alkyne click chemistry (green). HSAT3-related satellite signals were detected by RNA-FISH (red). Enlarged images of selected cells exposed to 42 °C for 1 h and corresponding fluorescence intensity profiles along the indicated white line are shown below. (**b**) Representative images of A549 and THP-1 cells exposed to 42 °C for 1 h in the presence of 5-EU. Newly synthesized RNA was visualized by click chemistry (green), and HSF1 was detected by immunofluorescence (red). Enlarged images of selected cells exposed to 42 °C for 1 h and corresponding fluorescence intensity profiles along the indicated white line are shown below. Nuclei were counterstained with DAPI (blue). Scale bars, 10 μm.

We analyzed nascent RNA distribution by 5-ethynyluridine (5-EU) incorporation under the same experimental conditions used to analyze HSF1 activation and HSAT3 transcription (Figure 1 and Figure 2). In control cells, EU labeling was predominantly distributed throughout DAPI-poor nuclear regions, consistent with transcriptionally active euchromatin. Following exposure to 42 °C for 1 h, the spatial distribution of nascent RNA changed in both cell lines, with extended DAPI-poor nuclear regions exhibiting low 5-EU signal intensity. In addition, distinct intense 5-EU-positive nuclear foci appeared in A549 cells, whereas comparable structures were not apparent in THP-1 cells (Figure 3a).

Combined 5-EU labeling and RNA-FISH using an HSAT3-targeting probe revealed an important methodological limitation. Control cells (37 °C) that were not incubated with 5-EU but were subjected to the click-reaction mixture subsequently displayed distinct HSAT3-positive nuclear signals, whereas no hybridization signal was detected in control cells analyzed by standard RNA-FISH without prior click-chemistry treatment (Figure S2). This indicates that the appearance of the HSAT3 signal was associated with the click-reaction conditions rather than with 5-EU incorporation. Because standard RNA-FISH does not include DNA denaturation, these findings suggest that components of the copper/ascorbate-based click-reaction mixture increased the accessibility of complementary genomic satellite sequences to the probe, potentially through partial local destabilization of DNA or chromatin structure [22]. Thus, under these conditions, the HSAT3 signal cannot be regarded as an exclusively RNA-specific readout and may include a contribution from accessible genomic DNA. This interpretation was further supported by RNase A pretreatment after the click-chemistry reaction, which did not abolish the HSAT3 hybridization signal detected by subsequent RNA-FISH (Figure 3a).

Nevertheless, in A549 cells exposed to 42 °C, prominent 5-EU-positive nuclear foci spatially coincided with HSAT3-positive satellite domains detected by the probe, and fluorescence intensity profile analysis demonstrated coincident enrichment of the two signals within these structures (Figure 3a). Because 5-EU marks newly synthesized RNA, this spatial association is consistent with active transcription occurring at or near HSAT3-positive pericentromeric satellite loci. In contrast, THP-1 cells did not exhibit prominent 5-EU-positive foci associated with HSAT3-positive domains after heat shock, and fluorescence intensity profiles showed little spatial correspondence between the two signals. However, because the HSAT3 probe signal obtained after click chemistry may contain a genomic DNA component, this experiment alone does not establish that the 5-EU-positive RNA within these foci consists specifically of HSAT3 transcripts or that the observed structures correspond to nSBs.

To further characterize these structures, we examined HSF1 localization by immunofluorescence, since HSF1 is a characteristic component of heat-induced nSBs. In heat-shocked A549 cells, the prominent 5-EU-positive nascent RNA foci showed pronounced spatial overlap with large HSF1-positive nuclear foci. In contrast, THP-1 cells did not show comparable discrete 5-EU-positive foci overlapping with HSF1 under the same heat-shock conditions (Figure 3b). Together with their association with HSAT3-related satellite domains, this observation supports the interpretation that the sites of intense nascent RNA accumulation detected in A549 cells are localized within HSF1-positive nuclear stress bodies. Importantly, this analysis does not require the assumption that the entire 5-EU-positive signal represents HSAT3 RNA; rather, it demonstrates active nascent RNA accumulation within HSF1-positive nuclear structures associated with HSAT3-related satellite domains.

Together, these observations indicate that heat shock is associated with the formation of discrete sites of nascent RNA accumulation in A549 cells that are associated with HSAT3-related pericentromeric satellite domains and overlap with HSF1-positive nSBs, whereas comparable structures are not detected in THP-1 cells.

### 2.4. HSAT3 Transcripts Colocalize with HSF1 During Nuclear Stress Body Assembly in A549 but Not THP-1 Cells

**Figure 4:**
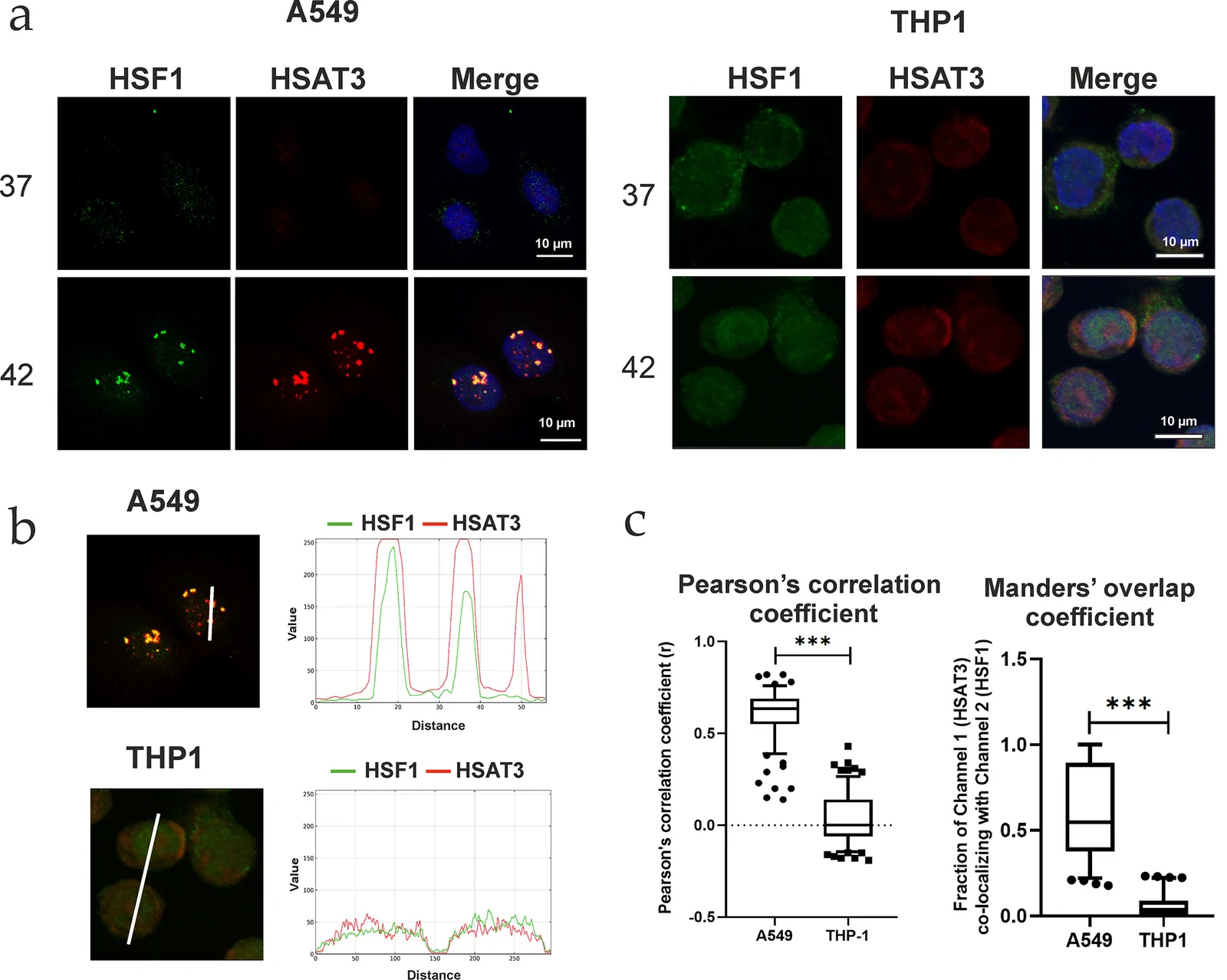
Heat shock-induced colocalization of HSAT3 transcripts with HSF1 in A549 but not THP-1 cells. (**a**) Representative images of A549 and THP-1 cells maintained at 37 °C or exposed to 42 °C for 1 h. HSAT3 transcripts detected by RNA-FISH are shown in red, HSF1 is shown in green, and nuclei are counterstained with DAPI (blue). Scale bars, 10 μm. (**b**) Representative line-scan profiles of HSF1 (green) and HSAT3 RNA-FISH (red) fluorescence intensities along the indicated white lines in heat-shocked A549 and THP-1 cells. (**c**) Pearson’s correlation coefficient (r) and thresholded Manders’ colocalization coefficient were calculated for individual nuclei after exposure to 42 °C for 1 h. The Manders’ coefficient represents the fraction of the HSAT3 signal overlapping with HSF1-positive regions and was calculated using Costes automatic thresholding. Boxes indicate the interquartile range, with the median shown as a horizontal line. *** *p* < 0.001.

To examine the spatial relationship between HSAT3 transcripts and HSF1 following heat shock, RNA-FISH was combined with HSF1 immunofluorescence in A549 and THP-1 cells exposed to 42 °C for 1 h. In A549 cells, heat shock induced the formation of 2–5 prominent HSAT3-positive nuclear foci accompanied by multiple smaller puncta (Figure 4a). The major HSAT3 foci showed pronounced spatial overlap with HSF1, consistent with nSB formation. This association was further supported by line-scan analysis, which demonstrated coincident HSAT3 and HSF1 fluorescence peaks within the major nuclear foci (Figure 4b). The median Pearson’s correlation coefficient calculated across individual cells was 0.635. To complement the Pearson analysis, the spatial overlap between HSAT3 and HSF1 was additionally assessed using a thresholded Manders’ coefficient with Costes automatic thresholding. Specifically, the fraction of the HSAT3 signal overlapping with HSF1-positive regions was quantified. The median Manders’ coefficient was 0.54 in A549 cells (Figure 4c). In contrast, THP-1 cells exhibited a dispersed pattern of HSAT3-positive signals throughout the nucleus after heat shock, without formation of prominent nuclear foci. HSF1 remained distributed as numerous small nuclear granules and did not accumulate in discrete nuclear bodies. Accordingly, line-scan profiles showed little or no spatial overlap between HSAT3 and HSF1 signals, and the median Pearson’s correlation coefficient was 0.00. Consistently, the median Manders’ coefficient was 0.03 in THP-1 cells, indicating minimal overlap of the HSAT3 signal with HSF1-positive regions (Figure 4c). To determine whether such structures might appear during the recovery period, we additionally examined THP-1 cells after 1 and 3 h of recovery following a 1 h heat shock at 42 °C. No detectable spatial overlap between HSAT3 and HSF1 was observed at either recovery time point, and prominent HSAT3/HSF1-positive nSBs were not detected (Figure S3). Thus, under the conditions examined, A549 cells formed HSAT3/HSF1-positive nSBs, whereas comparable structures were not detected in THP-1 cells despite the presence of nuclear HSAT3 signals. Although nSB formation has been studied predominantly in HeLa cells, similar heat-induced structures have also been reported in other epithelial tumor cell lines, including HCT-15 and ZR-75-1 [23]. To determine whether the response observed in A549 cells was reproduced in an additional epithelial model, we examined SK-BR-3 cells. Following exposure to 42 °C for 1 h, RNA-FISH using the HSAT3 probe revealed prominent nuclear foci that spatially overlapped with HSF1 immunofluorescence signals (Figure S4), consistent with nSB formation. Together with previous observations in other epithelial cell lines, these findings support a recurrent heat-induced HSAT3/HSF1-positive nSB response across epithelial tumor cell models.

### 2.5. Differentiation of THP-1 Cells Enables Heat-Induced Nuclear Stress Body Assembly

**Figure 5:**
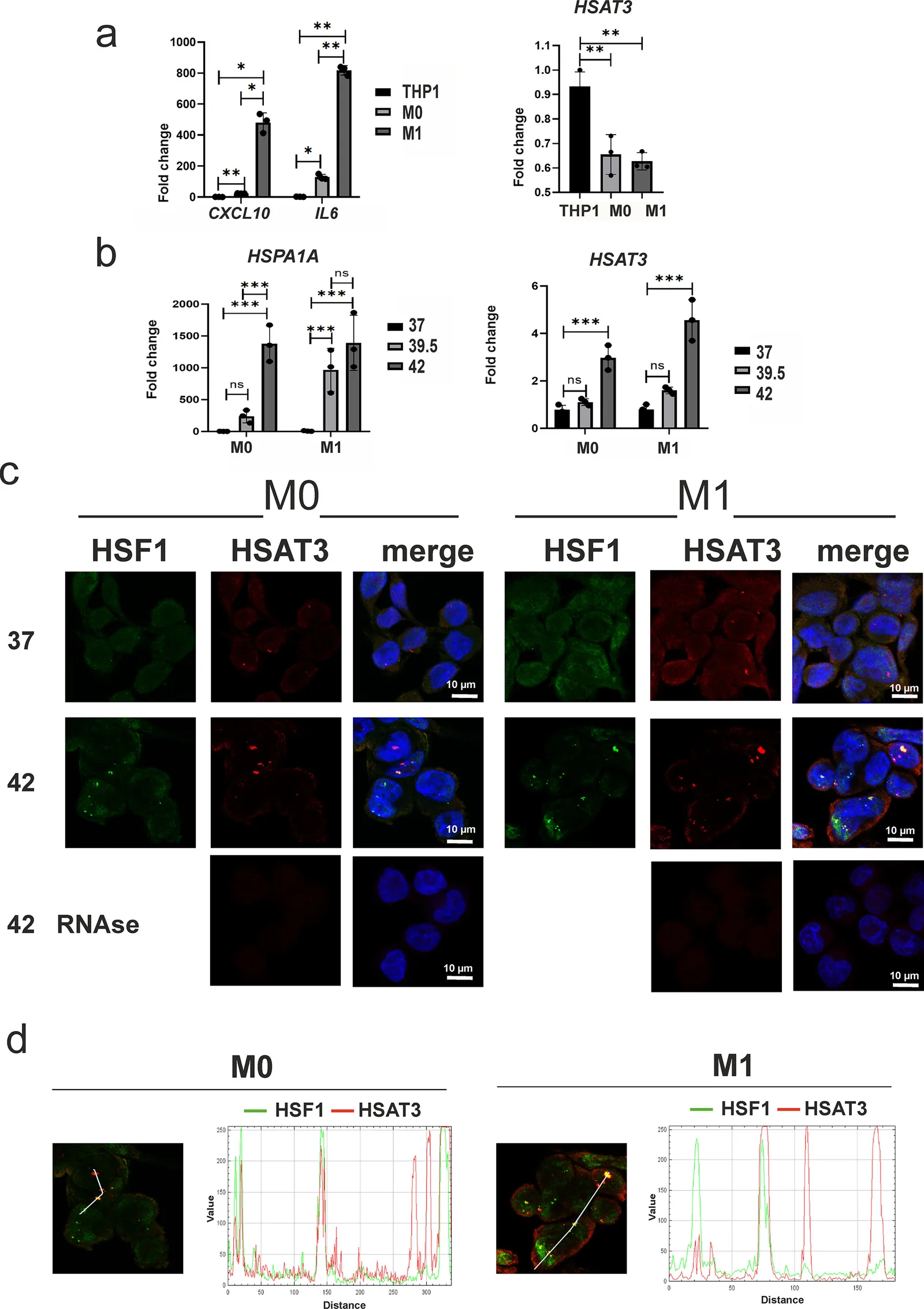
Effect of macrophage-like differentiation on heat-induced HSAT3 transcription and nuclear stress body formation in THP-1 cells. (**a**) Relative transcript levels of the pro-inflammatory marker genes *CXCL10* and *IL6* in undifferentiated THP-1 cells and THP-1-derived M0- and M1-like macrophages maintained at 37 °C. Relative HSAT3 transcript levels in the same cell populations under control conditions (37 °C) are shown on the right. (**b**) Relative *HSPA1A* and HSAT3 transcript levels in M0- and M1-like macrophages maintained at 37 °C or exposed to 39.5 or 42 °C for 1 h. Transcript levels were determined by qPCR and are presented as fold changes relative to the corresponding control. (**c**) Representative images of combined HSAT3 RNA-FISH (red) and HSF1 (green) immunofluorescence in M0- and M1-like macrophages maintained at 37 °C or exposed to 42 °C for 1 h. Additional representative HSAT3 RNA-FISH images of M0- and M1-like macrophages exposed to 42 °C for 1 h and treated with RNase A before hybridization are shown as specificity controls. Nuclei were counterstained with DAPI (blue). Scale bars, 10 μm. (**d**) Representative line-scan profiles of HSF1 (green) and HSAT3 (red) fluorescence intensities along the indicated white lines in M0- and M1-like macrophages following heat shock at 42 °C for 1 h. Data in (**a**,**b**) are presented as mean ± SD. * *p* < 0.05, ** *p* < 0.01, *** *p* < 0.001; ns, not significant.

To determine whether macrophage-like differentiation alters heat-induced HSAT3 transcription, THP-1 cells were differentiated into M0-like macrophages by PMA treatment, which resulted in the acquisition of an adherent macrophage-like morphology, and were subsequently polarized to an M1-like phenotype. The acquisition of a pro-inflammatory M1-like phenotype was supported by increased transcript levels of *CXCL10* and *IL6* (Figure 5a). In cells maintained at 37 °C, HSAT3 transcript levels were significantly lower in both M0- and M1-like cells than in undifferentiated THP-1 cells. No significant difference in HSAT3 transcript levels was observed between M0- and M1-like cells (Figure 5a). Consistent with the results obtained in undifferentiated THP-1 cells, heat shock induced *HSPA1A* expression in both M0- and M1-like macrophages, with increased transcript levels observed at 42 °C. However, in contrast to undifferentiated THP-1 cells, HSAT3 transcript levels increased significantly during heat shock at 42 °C in both M0- and M1-like macrophages (Figure 5b). Because HSAT3 transcript levels also increased in undifferentiated THP-1 cells after 3 h of recovery, we directly compared these levels with those observed in M0- and M1-like macrophages following heat shock at 42 °C using a common reference scale. HSAT3 levels in undifferentiated THP-1 cells after 3 h of recovery were comparable to those in heat-shocked M1-like macrophages and higher than in heat-shocked M0-like macrophages (Figure S5). Because increased HSAT3 levels in undifferentiated THP-1 cells during recovery were not accompanied by detectable HSAT3–HSF1 colocalization, we next examined nSB formation in macrophage-like THP-1 cells during heat shock.

The localization of HSAT3 transcripts and HSF1 was therefore analyzed using a combination of RNA-FISH and immunofluorescence. In control cells maintained at 37 °C, HSF1 and HSAT3 signals were distributed diffusely throughout the nucleus in both macrophage phenotypes (Figure 5c). Following heat shock at 42 °C, distinct HSAT3-positive nuclear foci became evident in both M0- and M1-like macrophages. These foci colocalized with HSF1 nuclear granules, consistent with the formation of nSBs. Line-scan analysis confirmed the overlap between HSAT3 and HSF1 fluorescence signals (Figure 5d). RNase A pretreatment strongly reduced the HSAT3 signal in heat-shocked M0- and M1-like macrophages, supporting the RNA-dependent nature of these foci (Figure 5c).

Collectively, these findings show that, in macrophage-like THP-1 cells, HSAT3 induction occurs during heat shock and is accompanied by the formation of HSAT3/HSF1-positive nSBs, whereas in undifferentiated THP-1 cells the increase in HSAT3 transcript levels is delayed until the recovery period and is not accompanied by detectable nSB formation.

To determine whether the differentiation-dependent HSAT3 response observed in THP-1 cells was also reproduced in another monocytic model, we examined PMA-differentiated U937 cells. Following macrophage-like differentiation, exposure to 42 °C resulted in increased HSAT3 transcript levels (Figure S6). These findings indicate that HSAT3 transcription induction during heat shock following macrophage-like differentiation is not restricted to THP-1 cells but is also observed in U937 cells.

## 3. Discussion

Heat-induced transcription of pericentromeric HSAT3 repeats and the assembly of nSBs have been described predominantly in epithelial and transformed human cell lines and are therefore commonly regarded as characteristic features of the cellular heat shock response. Whether this response is conserved in cells of non-epithelial origin, however, has remained largely unexplored. The present study indicates that this response is not universally conserved across human cell types. Whereas A549 cells showed a rapid and marked increase in HSAT3 transcript levels and assembled nSBs in response to heat shock, undifferentiated THP-1 cells showed no increase in HSAT3 transcript levels immediately after heat shock despite a clear canonical heat-shock response, as evidenced by increased pHSF1 (Ser326) levels and induction of *HSPA1A*. A delayed increase in HSAT3 transcript levels was observed only during the recovery period and was not accompanied by detectable nSB formation. Notably, differentiation into macrophages was associated with earlier HSAT3 induction during heat shock, and this response was retained following pro-inflammatory polarization. These findings identify differentiation status as an important determinant of stress-induced HSAT3 transcription in cells of the myeloid lineage.

Unlike promoters of canonical heat shock genes, HSAT3 arrays are embedded within constitutive pericentromeric heterochromatin. Several studies have shown that activation of these loci is accompanied by extensive chromatin remodeling, including histone acetylation, DNA demethylation and recruitment of chromatin-associated factors, suggesting that chromatin accessibility contributes to efficient HSAT3 transcription [12,13,17]. The chromatin environment therefore represents an additional regulatory layer controlling stress-induced satellite transcription. Differentiation of monocytes into macrophages is accompanied by extensive epigenetic remodeling, including changes in chromatin accessibility, histone modifications, DNA methylation, enhancer activity, and higher-order genome organization [24,25]. Although the chromatin organization of HSAT3 loci has not been examined directly during this process, these genome-wide changes provide a plausible explanation for the observed phenotype. Increased accessibility of pericentromeric regions may facilitate HSF1 binding or the recruitment of additional transcriptional regulators required for efficient HSAT3 transcription. An additional interesting feature of the THP-1 response was an apparent inverse relationship between basal HSAT3 expression and heat inducibility. Undifferentiated THP-1 cells displayed weak constitutive HSAT3-positive puncta and higher basal HSAT3 transcript levels than differentiated cells yet showed little immediate transcriptional response to heat shock. Differentiation reduced basal HSAT3 expression but was accompanied by earlier heat-induced HSAT3 transcription and the ability to form HSAT3/HSF1-positive nSBs. A similar differentiation-associated decrease in basal pericentromeric satellite transcription was observed in our previous study of primary human monocyte-derived macrophages, in which the levels of pericentromeric satellite transcripts declined during differentiation from monocytes to macrophages [16]. Evidence from both human and mouse cells indicates that pericentromeric satellite RNAs can contribute to the organization and maintenance of constitutive heterochromatin. In human cells, α-satellite RNAs remain associated in cis with pericentromeric regions and interact with the H3K9 methyltransferase SUV39H1, thereby contributing to its stable association with heterochromatin [26]. In mouse cells, major satellite repeat RNAs remain chromatin-associated and contribute to the stable association of Suv39h1/2 with heterochromatin, potentially through an RNA–nucleosome scaffold involving RNA:DNA hybrid formation [27]. These findings support the broader concept that basal satellite transcription can contribute to the maintenance of a repressive pericentromeric chromatin environment. It is therefore conceivable that relatively elevated basal HSAT3 transcription in undifferentiated THP-1 cells contributes to a regulatory state that limits further acute activation following heat shock. However, whether such an RNA-dependent feedback mechanism operates at human HSAT3 loci remains unknown and will require direct experimental investigation. Alternatively, differentiation may alter the abundance or nuclear localization of proteins participating in nSB assembly. Discriminating between these possibilities will require direct analysis of chromatin accessibility, HSF1 occupancy, and nSB composition during myeloid differentiation.

Our findings further suggest that nSB assembly is not a universal property of human cells but is shaped by cellular identity. Whether nSBs formed in different cellular contexts perform identical or distinct regulatory roles remains an important question for future investigation.

The differentiation-dependent activation of HSAT3 may reflect the adaptation of macrophages to their physiological environment. Unlike circulating monocytes, tissue-resident macrophages are long-lived cells that continuously sense and adapt to tissue-specific microenvironments. During inflammation and tissue injury, these cells are exposed to diverse stress-associated cues, including inflammatory mediators, oxidative stress, metabolic alterations, and hypoxia [28,29]. The capacity to activate stress-induced satellite transcription may therefore constitute an additional mechanism supporting nuclear homeostasis during prolonged or repeated stress. Whether this response contributes directly to macrophage survival, inflammatory signaling, or transcriptional adaptation remains to be determined.

Our results differ from those recently reported by Liu et al. [21], who observed heat-induced HSAT3 accumulation and nSB assembly in THP-1 cells. One factor that may account for this discrepancy is the well-recognized phenotypic heterogeneity of THP-1 cultures. Passage number, serum composition, culture density, and basal activation state influence this cell line’s transcriptional and epigenetic landscape [30]. Because even partial spontaneous differentiation may alter chromatin organization, variability in the differentiation status of THP-1 cultures could substantially affect their capacity to activate HSAT3 transcription following heat shock.

Beyond their physiological relevance, the present findings may have implications for cancer biology. HSF1 is frequently activated in human tumors through multiple oncogenic and stress-associated signaling pathways and promotes malignant progression by regulating transcriptional programs extending beyond the classical heat shock response [31,32]. Elevated HSF1 activity, however, is generally not accompanied by constitutive HSAT3 transcription or persistent nSB assembly, indicating that satellite transcription requires additional regulatory conditions. Consistent with this notion, Kanne et al. [33] demonstrated that heat-induced HSAT3 transcripts promote resistance to etoposide by recruiting TOP2A to nSBs, thereby limiting DNA damage.

In conclusion, our study demonstrates that stress-induced HSAT3 transcription is not a universal consequence of heat shock but a context-dependent response determined by cellular differentiation. These findings expand the current understanding of HSAT3 regulation and establish myeloid differentiation as an important factor governing the activation of pericentromeric satellite transcription and nSB assembly during heat shock.

## 4. Materials and Methods

### 4.1. Cell Culture

The human lung adenocarcinoma cell line A549 and the human monocytic cell lines THP-1 and U937 were obtained from the Russian Collection of Cell Cultures (Institute of Cytology, Russian Academy of Sciences, St. Petersburg, Russia; collection catalog: https://cellcollection.ru/catalog/ (accessed on 11 October 2026); the cell lines are catalogued under their standard designations A549, THP-1, and U937). The SK-BR-3 human breast adenocarcinoma cell line was obtained from the American Type Culture Collection (ATCC, Manassas, VA, USA; HTB-30). A549 cells were cultured in low-glucose Dulbecco’s Modified Eagle Medium (DMEM; 1 g/L glucose; Gibco, Thermo Fisher Scientific, Waltham, MA, USA), SK-BR-3 cells in DMEM/F12 (PanEco, Moscow, Russia), and THP-1 and U937 cells in RPMI-1640 medium (Thermo Fisher Scientific, Waltham, MA, USA). All culture media were supplemented with 10% fetal bovine serum (FBS), 100 U/mL penicillin, and 100 μg/mL streptomycin (Life Technologies, Carlsbad, CA, USA). Cells were maintained at 37 °C in a humidified atmosphere containing 5% CO_2_. Before experiments, cells were passaged and maintained under standard culture conditions for 24 h. Heat shock was induced by incubating the cells in a water bath for 1 h at 39.5, 42, or 45 °C. Following heat shock, cells were analyzed immediately. For additional time-course experiments, THP-1 cells were exposed to 42 °C for 1 or 2 h. To examine the post-stress recovery period, THP-1 cells were exposed to 42 °C for 1 h and either analyzed immediately (0 h recovery) or returned to 37 °C for 1 or 3 h of recovery before analysis. Control samples underwent the same handling procedures without exposure to elevated temperatures.

### 4.2. Differentiation of THP-1 and U937 Cells and Macrophage Polarization

THP-1 and U937 cells were seeded at a density of 2 × 10^5^ cells/mL. After 24 h, phorbol 12-myristate 13-acetate (PMA; Sigma-Aldrich, St. Louis, MO, USA) was added to the culture medium at a final concentration of 50 ng/mL to induce differentiation into adherent M0-like macrophages. To generate M0-like macrophages, cells were incubated with PMA for 48 h. For M1 polarization, the culture medium was replaced with fresh medium after 24 h of PMA treatment and supplemented with interferon-γ (IFN-γ; 100 ng/mL; ImmunoTools GmbH, Friesoythe, Germany) and lipopolysaccharide (LPS; 200 ng/mL; Servicebio, Wuhan, China). Cells were then incubated under these conditions for an additional 24 h.

### 4.3. Western Blot Analysis

Following the experimental treatment, cells were lysed in a buffer containing 25 mM Tris-HCl (pH 6.8), 2% SDS, 10% glycerol, and 5% β-mercaptoethanol, and the lysates were heated at 100 °C for 10 min. Proteins were separated by 10% SDS-PAGE and transferred to polyvinylidene difluoride (PVDF) membranes (Bio-Rad Laboratories, Hercules, CA, USA). Membranes were blocked with 5% bovine serum albumin (BSA) in TBST and incubated with rabbit anti-phospho-HSF1 (Ser326) antibody (Abcam, Cambridge, UK; 1:2000) and mouse anti-α-tubulin antibody (Sigma-Aldrich, St. Louis, MO, USA). α-Tubulin was used as a loading control. Membranes were washed three times for 10 min each with TBST, then incubated with the corresponding alkaline phosphatase (AP)-conjugated anti-rabbit and anti-mouse secondary antibodies (Sigma-Aldrich, St. Louis, MO, USA) at dilutions of 1:5000 and 1:10,000, respectively. Immunoreactive bands were visualized using the BCIP/NBT substrate system. Membranes were imaged, and band intensities were quantified by densitometric analysis using TotalLab 120 1D v2009 software (Nonlinear Dynamics Ltd., Newcastle upon Tyne, UK).

### 4.4. RNA Extraction and Reverse Transcription

Total RNA was extracted from cells using ExtractRNA reagent (Evrogen, Moscow, Russia) according to the manufacturer’s instructions. To remove potential genomic DNA contamination, RNA samples were treated with DNase I (Fermentas, Waltham, MA, USA) for 30 min at 37 °C. DNase I was inactivated by addition of EDTA followed by incubation at 65 °C for 5 min. Reverse transcription was performed using the MMLV Reverse Transcriptase kit (Evrogen, Moscow, Russia) with 500 ng of total RNA per reaction. cDNA was synthesized using either oligo(dT) or the HSAT3-specific RSM13 primer for G-rich transcripts, as previously described by Valgardsdottir et al. [5]. The RSM13 primer contained a 5′ M13 adapter sequence and had the following sequence: 5′-CCGTAAAACGACGGCCAGTTCCCTTCCATTCCATTATTATCC-3′. For selective amplification of HSAT3 transcripts, a strand-specific HSAT3 primer was used in combination with the universal M13 primer, enabling selective amplification of HSAT3-derived cDNA while minimizing amplification from residual genomic DNA.

### 4.5. qPCR

Quantitative PCR (qPCR) was performed using 5X qPCRmix-HS SYBR reagent (Evrogen, Moscow, Russia) on a CFX96 Touch Real-Time PCR Detection System (Bio-Rad Laboratories, Inc., Hercules, CA, USA). Amplification was performed using the following primers: *HSPA1A*: forward 5′-CGTGTTGGAACACCCCCACG-3′, reverse 5′-TTCCGTTTCCAGCCCCCAATC-3′; *CXCL10*: forward 5′-TGGCATTCAAGGAGTACCTCTC-3′, reverse 5′-TGATGGCCTTCGATTCTGGA-3′; *IL6*: forward 5′-AACTCCTTCTCCACAAGCGCC-3′, reverse 5′-GTGGGGCGGCTACATCTTT-3′; *GAPDH*: forward 5′-AGGTCGGAGTCAACGGATTT-3′, reverse 5′-TTCCCGTTCTCAGCCTTGAC-3′. For HSAT3 quantification, cDNA generated using the RSM13 primer containing the 5′ M13 adapter was amplified with an HSAT3-specific primer (5′-AATCAACCCGAGTGCAATCG-3′) and the universal M13 primer (5′-CCGTAAAACGACGGCCAG-3′). This strategy preferentially amplifies HSAT3-derived cDNA and reduces amplification from residual genomic DNA. *GAPDH* transcript stability under the experimental conditions, including heat shock and macrophage differentiation, was verified prior to the use of *GAPDH* as the reference gene for relative quantification. The amplification protocol consisted of an initial denaturation at 95 °C for 5 min, followed by 40 cycles of 95 °C for 15 s, 60 °C for 15 s, and 72 °C for 30 s. Amplification specificity was assessed by melting-curve analysis. Relative transcript levels were calculated using the comparative Ct (2^−ΔΔCt^) method.

### 4.6. Fluorescence In Situ Hybridization and Immunocytochemistry

For fluorescence in situ hybridization (FISH), cells were washed with PBS to remove the culture medium and fixed with 10% formalin containing 0.02% Tween-20 for 15 min. The samples were then washed twice with PBS, followed by two washes with 2× saline-sodium citrate (SSC) buffer, and air-dried. A hybridization mixture containing Hybridozol VII (MP Biomedicals, Irvine, CA, USA) and a Cy3-labeled single-stranded DNA probe targeting HSAT3 (DYZ1 probe) 5′-TCCATTCCATTCCATTCCATTCCATTCCATTCCATTCCATTCCATTCC-3′ [20] was applied to the samples. The DYZ1 probe, composed of ATTCC repeats, is complementary to the GGAAT-rich strand of HSAT3 and therefore detects G-rich HSAT3 transcripts, which represent the predominant heat-induced SatIII transcripts. Hybridization was performed overnight at 37 °C. Following hybridization, the samples were washed sequentially in SSC buffers of decreasing concentration (2×, 1×, 0.5×, and 0.25× SSC) for 10 min each. The 2× SSC wash was performed at 41 °C for 10 min, whereas subsequent washes were performed at room temperature. Samples were then rinsed with distilled water and mounted using an antifade mounting medium containing DAPI (Lumiprobe, Moscow, Russia). For RNase controls, fixed cells were treated with RNase A (100 µg/mL) for 1 h at 37 °C before hybridization. After treatment, the cells were washed with 2× SSC and subjected to RNA-FISH under the same conditions as untreated samples. For combined FISH and immunocytochemical staining, samples were processed for immunostaining following the final 0.25× SSC wash. Samples were washed with PBS and blocked with 5% bovine serum albumin (BSA) in PBS for 1 h. They were then incubated with a rat anti-HSF1 primary antibody (Santa Cruz Biotechnology, Dallas, TX, USA; 1:100) for 1 h, followed by three 10-min washes with PBS. Samples were subsequently incubated with a FITC-conjugated anti-rat secondary antibody (Sigma-Aldrich, St. Louis, MO, USA; 1:100) for 1 h and washed three times with PBS for 10 min each. Finally, samples were rinsed with distilled water and mounted using an antifade medium containing DAPI (Lumiprobe, Moscow, Russia).

### 4.7. 5-EU Labeling and Detection of Nascent RNA

To assess nascent RNA synthesis during heat shock, the culture medium was replaced immediately before heat treatment with fresh medium supplemented with 1 mM 5-ethynyluridine (5-EU) (Lumiprobe, Moscow, Russia). Cells were incubated under heat-shock conditions for 1 h and subsequently washed with PBS and fixed with 10% formalin containing 0.02% Tween-20 for 15 min at room temperature. Following fixation, cells were washed with TBS and subjected to click-chemistry labeling to detect incorporated 5-EU. The click-reaction mixture was prepared in 100 mM Tris-HCl (pH 8.5) and contained 1 mM CuSO_4_, 10 μM azide-FITC (Lumiprobe, Moscow, Russia), and 100 mM ascorbic acid. Cells were incubated with the reaction mixture for 30 min at room temperature. Following the reaction, cells were washed with TBS containing 0.02% Tween-20 and either processed for subsequent in situ hybridization as described above or mounted in an antifade medium containing DAPI (Lumiprobe, Moscow, Russia).

### 4.8. Confocal Microscopy and Image Analysis

Fluorescence images were acquired using an Olympus FV3000 confocal laser scanning microscope (Olympus Corporation, Tokyo, Japan) equipped with 405, 488, and 561 nm lasers for excitation of DAPI, FITC, and Cy3, respectively. Images were acquired using a 40× objective. Z-stack images were collected with a step size of 0.8 μm. At least five randomly selected fields of view were acquired for each sample. Imaging parameters were optimized separately for each cell line. Within each cell type, corresponding control and heat-shocked samples were acquired using identical imaging settings. Absolute fluorescence intensities were not compared between A549 and THP-1 cells. Image acquisition and processing were performed using the Olympus imaging software FV31S-SW v2.6 (Olympus Corporation, Tokyo, Japan) and Fiji (ImageJ, version 1.54p; National Institutes of Health, Bethesda, MD, USA). Colocalization of fluorescence signals was assessed using Pearson’s correlation coefficient and a thresholded Manders’ coefficient with Costes automatic thresholding, with the latter representing the fraction of the HSAT3 signal overlapping with HSF1-positive regions. In addition, fluorescence intensity profiles were generated to evaluate the spatial distribution and overlap of signals between channels.

### 4.9. Statistical Analysis

Data were obtained from at least three independent biological experiments. For quantitative PCR, each biological sample was analyzed in three technical replicates, which were averaged prior to statistical analysis. Statistical analyses were performed using GraphPad Prism version 9.0.2 (GraphPad Software, San Diego, CA, USA). Differences in transcript levels among multiple experimental groups were assessed using one-way analysis of variance (ANOVA) followed by Dunnett’s multiple comparisons test, with the corresponding control group used as the reference. A *p* value < 0.05 was considered statistically significant. For quantitative analysis of RNA-FISH signals, three randomly selected fields of view were analyzed for each sample, with approximately 30–60 nuclei evaluated per field. The fraction of nuclear area occupied by the RNA-FISH signal was quantified for individual nuclei. Differences between two groups, including comparisons of Pearson’s correlation and Manders’ coefficients, were assessed using an unpaired two-tailed Mann–Whitney U test. RNA-FISH data and Pearson’s correlation coefficients are presented as individual values with the median and interquartile range.

## 5. Conclusions

Rather than representing a universal consequence of the canonical heat-shock response, the HSAT3-dependent pathway emerges as a context-dependent branch of the heat shock response, with activation determined by cellular identity and differentiation state. The main limitations of this study are the use of established cell lines, the limited phenotypic characterization of THP-1-derived M1-like macrophages without analysis of specific surface markers, the lack of direct analysis of chromatin accessibility and HSF1 occupancy at HSAT3 loci, and the potential contribution of genomic DNA to the DYZ1 signal in the combined 5-EU/RNA-FISH experiments.

## Data Availability

The original contributions presented in this study are included in the article/Supplementary Materials. Further inquiries can be directed to the corresponding authors.

## Supplementary Materials

The following supporting information can be downloaded at: https://www.mdpi.com/article/10.3390/ijms27209146/s1.

## Abbreviations

The following abbreviations are used in this manuscript:

5-EU	5-ethynyluridine
AP	alkaline phosphatase
cDNA	complementary DNA
DAPI	4′,6-diamidino-2-phenylindole
FISH	fluorescence in situ hybridization
HSAT2	human satellite 2
HSAT3	human satellite 3
HSF1	heat shock factor 1
HSP70	heat shock protein 70
IFN-γ	interferon gamma
LPS	Lipopolysaccharide
nSB	nuclear stress body
pHSF1	phosphorylated heat shock factor 1
PMA	phorbol 12-myristate 13-acetate
PVDF	polyvinylidene difluoride
qPCR	quantitative polymerase chain reaction
RNA-FISH	RNA fluorescence in situ hybridization
RNP	Ribonucleoprotein
SD	standard deviation
SDS-PAGE	sodium dodecyl sulfate–polyacrylamide gel electrophoresis
SSC	saline–sodium citrate
TBS	Tris-buffered saline
TBST	Tris-buffered saline containing Tween-20
