# Supplementary material for: Heat-Induced HSAT3 Transcription and Nuclear Stress Body Assembly in Human Myeloid Cells
Source: Int J Mol Sci. 2026 Oct 15;27(20):9146. doi: 10.3390/ijms27209146 (PMC13705371; doi:10.3390/ijms27209146)
Supplement: Supplementary file 1 [file ijms-27-09146-s001.zip › ijms-4593030-supplementary_finish.pdf]

SUPPLEMENTARY MATERIALS

Heat-Induced HSAT3 Transcription and Nuclear Stress Body Assembly in Human Myeloid Cells

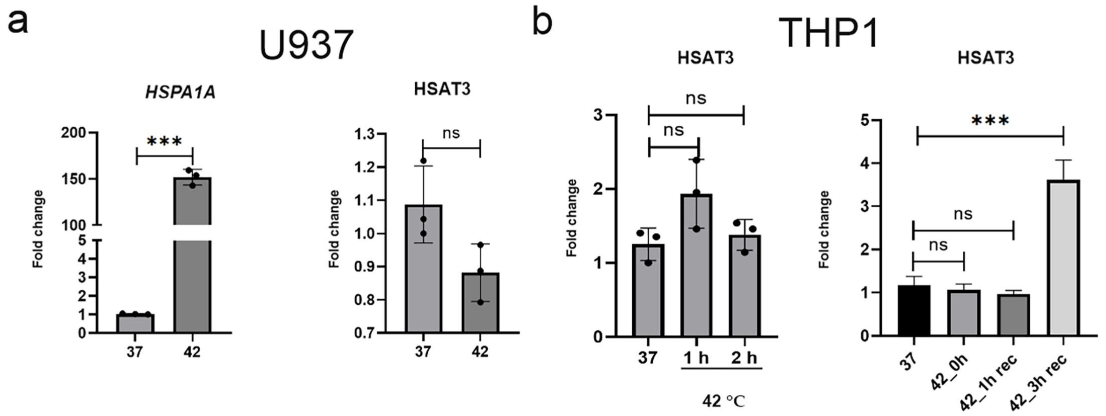

Figure S1. Heat-shock response in U937 cells and time-course analysis of HSAT3 expression in THP-1 cells. **(a)** Relative *HSPA1A* and HSAT3 transcript levels were analyzed by qPCR in undifferentiated U937 cells maintained at 37 °C or exposed to 42 °C for 1 h. (b) Time-course analysis of HSAT3 expression in undifferentiated THP-1 cells during heat exposure and the recovery period. HSAT3 transcript levels were analyzed by qPCR in cells maintained at 37 °C, exposed to 42 °C for 1 or 2 h, or exposed to 42 °C for 1 h and analyzed immediately after heat shock (0 h recovery) or after 1 or 3 h of recovery at 37 °C. Expression levels are presented as fold changes relative to the corresponding 37 °C control. Data are presented as mean ± SD. Statistical significance was assessed using one-way ANOVA. *** $p < 0.001$; ns, not significant.

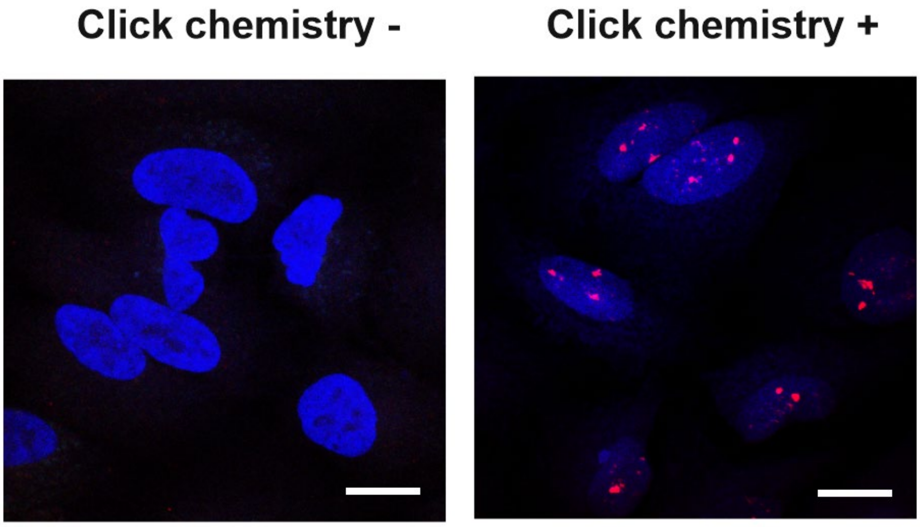

Figure S2. Effect of click-reaction conditions on HSAT3 probe hybridization during RNA-FISH. Representative RNA-FISH images of control cells maintained at 37 °C and either not exposed to the click-reaction mixture (Click chemistry −) or incubated with the click-reaction mixture used for Cu(I)-catalyzed azide–alkyne cycloaddition (Click chemistry +). Cells were not incubated with 5-EU. HSAT3-related satellite signals were detected by RNA-FISH using the HSAT3 probe and are shown in red; nuclei were counterstained with DAPI (blue). Scale bars, 10 μm.

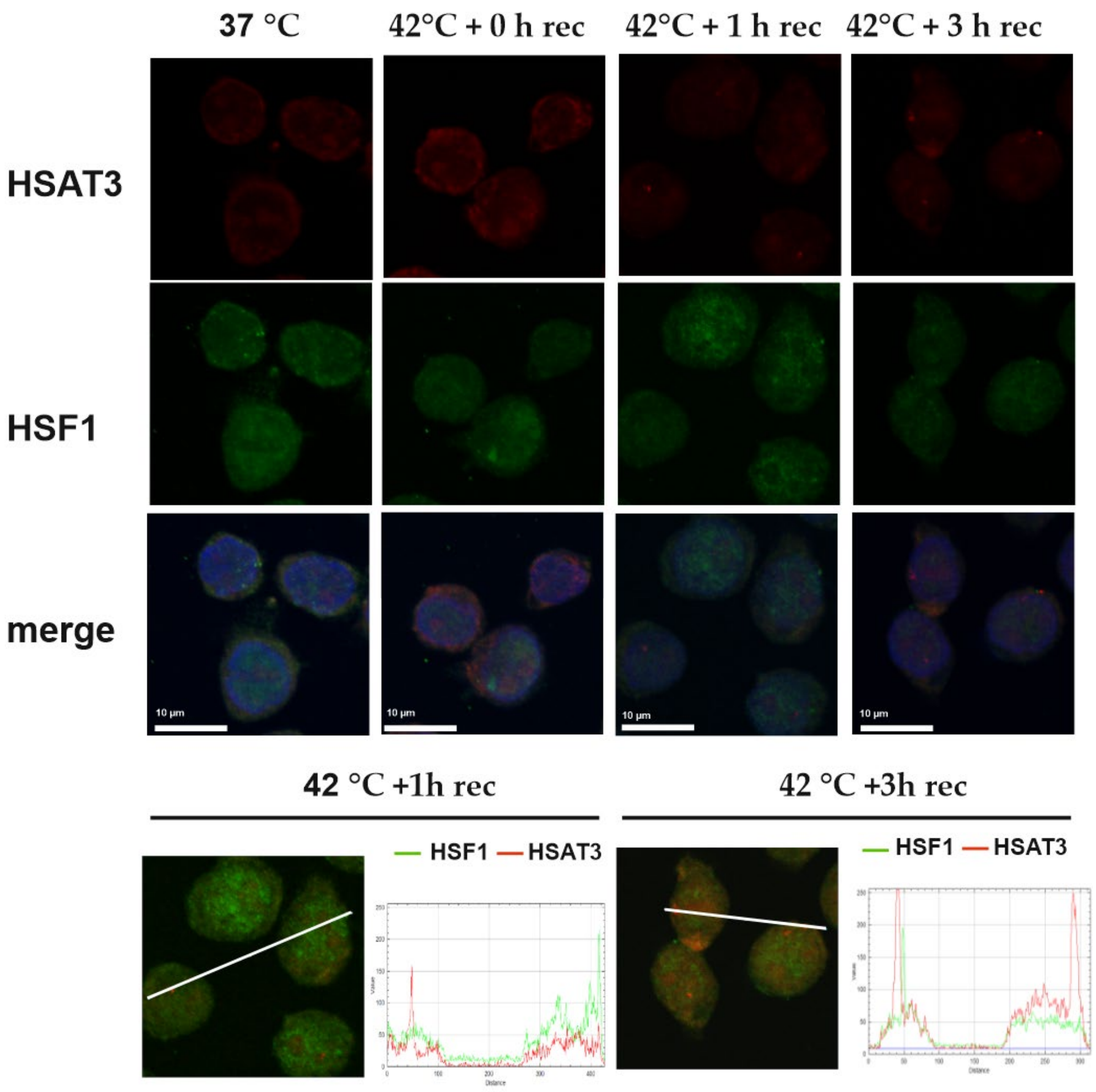

Figure S3. Lack of detectable HSAT3–HSF1 colocalization in THP-1 cells during the recovery period. Representative images of undifferentiated THP-1 cells maintained at 37 °C or exposed to 42 °C for 1 h and analyzed immediately after heat shock (0 h recovery) or after 1 or 3 h of recovery at 37 °C. HSAT3 transcripts detected by RNA-FISH are shown in red, HSF1 is shown in green, and nuclei are counterstained with DAPI (blue). Representative line-scan profiles of HSF1 (green) and HSAT3 RNA-FISH (red) fluorescence intensities along the indicated white lines are shown for cells after 1 and 3 h of recovery. Scale bars, 10 µm.

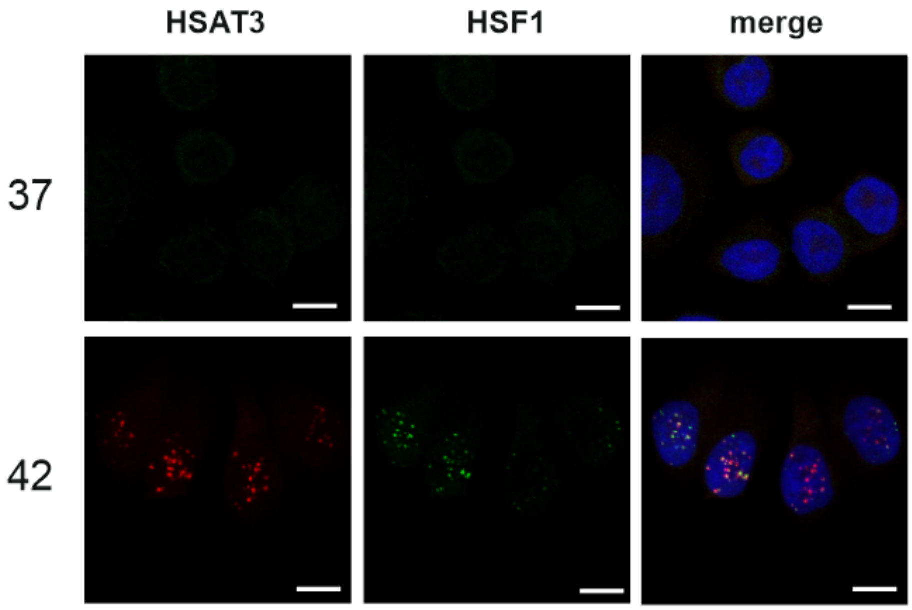

Figure S4. Heat shock-induced colocalization of HSAT3 transcripts with HSF1 in SK-BR-3 cells. Representative images of SK-BR-3 cells maintained at 37 °C or exposed to 42 °C for 1 h. HSAT3 transcripts detected by RNA-FISH are shown in red, HSF1 is shown in green, and nuclei are counterstained with DAPI (blue). Scale bars, 10 µm.

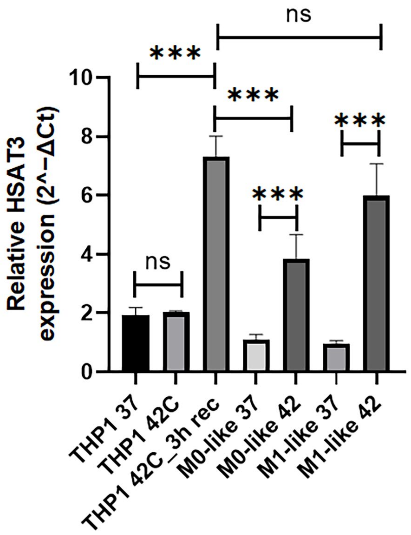

Figure S5. HSAT3 transcript levels in undifferentiated and macrophage-like THP-1 cells on a common reference scale. Relative HSAT3 transcript levels was measured in undifferentiated THP-1 cells under control conditions, after 1 h at 42 °C, and after 3 h of recovery following heat shock, as well as in M0- and M1-like macrophages maintained at 37 °C or exposed to 42 °C for 1 h. Expression values are presented as $2^{-\Delta Ct}$, with *GAPDH* used as the reference gene. Statistical comparisons were performed using ΔCt values. Data are presented as mean ± SD. *** $p < 0.001$; ns, not significant.

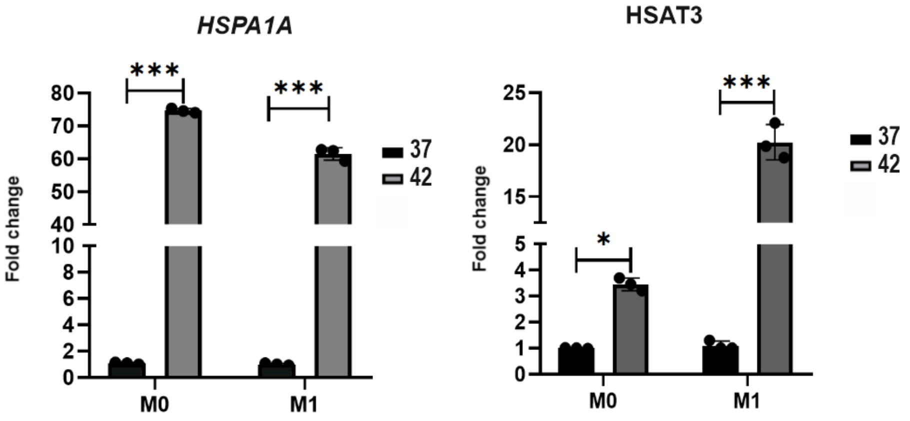

Figure S6. Heat-induced activation of HSAT3 transcription in U937-derived macrophages. Relative expression levels of *HSPA1A* and HSAT3 in U937-derived M0- and M1-like macrophages maintained at 37 °C or exposed to 42 °C for 1 h. Data are presented as mean ± SD. * $p < 0.05$, *** $p < 0.001$; ns, not significant.
